# Accuracy of endobronchial ultrasound-guided transbronchial needle aspiration in HIV-infected patients with thoracic lymphadenopathy in a tuberculosis low-burden area

**DOI:** 10.1038/s41598-020-73153-6

**Published:** 2020-10-01

**Authors:** Macha Tetart, Farid Betraoui, Thomas Huleux, Frédéric Wallyn, Anne Brichet, Pauline Thill, Olivier Robineau, Agnès Meybeck

**Affiliations:** 1 Service Des Maladies Infectieuses, Centre Hospitalier de Tourcoing, 135 Avenue du Président Coty, 59200 Tourcoing, France; 2 Service de Pneumologie, Centre Hospitalier de Tourcoing, Tourcoing, France; 3 Service D’Endoscopie Bronchique, Centre Hospitalier Régional Universitaire de Lille, Lille, France; 4 Service de Pneumologie, Centre Hospitalier de Roubaix, Roubaix, France

**Keywords:** Diseases, Health care, Medical research

## Abstract

Endobronchial ultrasound-guided transbronchial needle aspiration (EBUS-TBNA) is an innovative technique to explore hilar and mediastinal lymphadenopathy. We aimed to assess its diagnostic accuracy in HIV-infected patients in a tuberculosis low-burden area. A retrospective review was performed of all HIV-infected patients with thoracic lymphadenopathy referred for EBUS-TBNA between January 2012 and January 2019 in 3 Northern French Hospitals. A total of 15 patients was included during the study period. Our patients were predominantly male (80%), with a mean age of 50 ± 11 years. Six patients (43%) had a CD4 cells count of less than 200/mm^3^. Eleven patients (73%) were receiving antiretroviral therapy, and 7 (47%) reached undetectable viral load. Adequate lymphnode sampling was accomplished in all patients. No serious complications were reported. EBUS-TBNA led to a definitive diagnosis in 12 out of 15 patients (80%). It identified 4 neoplasia, 3 atypical mycobacterial diseases, 2 tuberculosis, 1 Castleman disease, 1 sarcoidosis, and 1 professional dustiness. In 3 cases, sampling revealed normal lymphoid tissue. Active surveillance confirmed the suspected diagnosis of HIV adenitis with regression of lymphadenopathy on antiretroviral therapy in 2 cases. In one case of negative sampling, thoracoscopy led to the diagnosis of tuberculosis. In our cohort, accuracy of EBUS-TBNA was 92%. EBUS-TBNA appeared to be a safe and accurate tool in the investigation of mediastinal lymphadenopathy in HIV-infected patients in settings of tuberculosis low-prevalence. It can avoid more invasive procedures such as mediastinoscopy.

## Introduction

Mediastinal and hilar lymphadenopathy is a common condition in patients infected with HIV.

It has been previously reported to occur in up to 35–40% of patients infected with HIV^1^. The broad range of aetiologies and the non-specific clinical presentation represent a diagnostic challenge^2,3^. In most of cases, histopathological samples are needed to establish diagnosis. Several techniques are available to obtain pathological samples of mediastinal or hilar lymphnodes including mediastinoscopy, computed tomography (CT) guided needle aspiration, and conventional bronchoscopic transbronchial needle aspiration (TBNA). TBNA is a minimally invasive and safe technique that allows sampling of mediastinal nodes, avoiding the use of more invasive procedures such as mediastinoscopy. TBNA proved to be usefull in HIV-infected patients with intrathoracic lymphadenopathy due to mycobacterial infection^4^. By integrating ultrasound technology into a flexible bronchoscope, endobronchial ultrasound (EBUS) is an innovative technique which allows accurate definition of mediastinal structures and enhances site selection for transbronchial sampling^5^. EBUS-TBNA has become an essential tool and is now one of the recommended first steps in the mediastinal staging of lung cancer^6^. It is also a reliable way to diagnose conditions such as sarcoidosis and tuberculosis^7,8^. Data concerning use of EBUS-TBNA in HIV-infected patients are limited. Few studies, all conducted in tuberculosis high or intermediate burden area reported case series of HIV-infected patients who underwent EBUS-TBNA and demonstrated high accuracy^9–11^. Majority of patients were diagnosed with tuberculosis. The authors assumed that the diagnostic yield of EBUS-TBNA in HIV patients, even in a TB low burden area would be high. We performed a retrospective review of all HIV-infected patients with thoracic lymphadenopathy referred for EBUS-TBNA in 3 Northern French Hospitals, a tuberculosis low-incidence area.

## Patients and methods

### Patients and hospital setting

We conducted a multicentre retrospective cohort study in 3 Northern French Hospitals (Lille, Roubaix, Tourcoing), over a period of 7 years (1 January 2012 through 1 January 2019). Lille hospital is an academic hospital. Both other hospitals are general hospitals. All consecutive HIV-infected patients with thoracic lymphadenopathy referred for EBUS-TBNA during the study period were retrospectively included. In all cases, EBUS-TBNA was indicated to establish the etiologic diagnosis of an enlarged lymph node of unknown cause. All the patients were evaluated by CT chest with contrast before the procedure, with or without positron emission tomography-computed tomography (PET/CT). Mediastinal or hilar lymph nodes that measure > 1 cm in short axis are considered as enlarged.

### Ethics

According to French Law, written informed consent was obtained for all patients who signed consent forms for participation to clinical database (Nadis) and later use of the data^12^. The computerized management of data was approved by the French Data Protection Authority (Commission nationale de l’informatique et des libertés (CNIL) 770,134). Our study was carried out in accordance with national guidelines concerning observational study conducted retrospectively on collected data (article R.1121-1-1, Décret n°2017-884 du 9 mai 2017). The present study get ethical approval from the local ethical committee of Dron Hospital (Comité d’éthique du Centre Hospitalier Gustave Dron).

### Data collection

Demographic data and characteristics of all patients who benefited from EBUS-TBNA were collected using clinical database (Nadis)^12^. Following data on HIV infection were recorded: year of HIV diagnosis, nadir of CD4 cell count, zenith of HIV-VL, HIV disease staging according to CDC Classification System for HIV-Infected Adults (Category A: asymptomatic, acute HIV, or persistent generalized lymphadenopathy; Category B: symptomatic conditions not A or C; Category C: AIDS-indicator conditions), the duration of undetectability, CD4 cell count and plasma HIV-VL at the time of EBUS-TBNA performance. History of antiretroviral treatment was collected: number of successive antiretroviral regimens, duration of antiretroviral therapy, and current antiretroviral treatment at the time of EBUS-TBNA performance. Following clinical data were recorded: other underlying clinical conditions, toxic habits, symptoms and clinical signs.

### EBUS-TBNA procedure

The seventh edition of the classification of the International Association for the Study of Lung Cancer (IASLC) was used to define lymph node stations: 1, highest mediastinal; 2, upper paratracheal; 3, pre-vascular and retrotracheal; 4, lower paratracheal; 5, subaortic; 6, para-aortic; 7, subcarinal; 8, paraesophageal; 9, pulmonary ligament; 10, hilar; 11, interlobar; 12, lobar; 13, segmental; 14, subsegmental.

All patients signed an informed consent before EBUS-TBNA performance. The procedure was performed under conscious sedation with midazolam and nebulized local anaesthesia with lidocaine 1%-2% using a convex probe echo-endoscope (BF-UC160F/180FOL8; Olympus, Tokyo, Japan). Lymph nodes were sampled with a 21-gauge Olympus Vizi Shot single-use aspiration needle, as previously described^8^. Rapid-on-site cytologic evaluation was not performed. Histopathological and microbiological examination was performed. Direct examination for acid-fast bacilli using Ziehl–Neelsen staining and mycobacterial culture were performed in all cases. The Xpert MTB/RIF assay was performed only in case of positive microscopy for acid-fast bacilli. EBUS-TBNA was deemed as diagnostic, if it led to specific diagnosis (tuberculosis, malignancy, lymphoma, sarcoidosis, or others), or if it showed normal lymphatic tissue and a follow-up of at least one year confirmed stability or improvement in patient’s condition, ruling out false-negative result.

### Statistical analysis

Continuous variables were expressed as mean and standard deviation. They were compared using the Mann–Whitney test. Categorical variables were expressed as number and percentage. They were compared using the Fisher’s exact test. Differences between groups were considered to be significant for variables yielding a p value < 0.05.

The primary end point was the percentage of confirmed diagnosis made possible with EBUS-TBNA. Diagnostic sensitivity, specificity, and accuracy were calculated using the standard definitions: the proportion of true positive results, true negative results, and all correct results. The unit of analysis was the patients.

### Ethics approval

All patients have signed an agreement allowing the record of their data in a database (NADIS) approved by a French data protection authority (Commission nationale de l’informatique et des libertés (CNIL) 770134). As a retrospective survey and in accordance with the European General Data Protection Regulation (n°2016/679) and the French Law (article R. 1121-1-1 du code de la santé publique, Décret n° 2017-884 du 9 mai 2017), this study has been locally registered in Dron Hospital and approved by the local ethical committee (comité d’éthique du Centre Hospitalier Gustave Dron).

## Results

### Study population

From January 2012 to December 2018, 15 HIV-infected patients underwent EBUS-TBNA. Only 4 procedures have been performed before 2015. Demographic data and characteristics of the patients are summarized in Table 1. Our patients were predominantly males (80%), with a mean age of 50 years. The mean time between HIV diagnosis and EBUS-TBNA performance was 14 ± 11 years. The majority of our patients were receiving antiretroviral treatment (73%), with a viral load (VL) below 20 copies/ml in 47%. The mean CD4 cells count was 345/mm^3^. Six patients (43%) had a CD4 cells count of less than 200/mm^3^.

**Table 1:** Demographic and clinical characteristics of the patients at the time of EBUS-TBNA performance.

Characteristics	Total n = 15
**Demographic characteristics**	
Age (years)	50 ± 11
Male sex	12 (80%)
**HIV infection**	
CDC stage C	6 (40%)
Nadir CD4 cell count (/mm^3^)	195 ± 162
Zenith viral load (copies/ml)	265 031 ± 325 260
CD4 cell count (/mm^3^)	345 ± 292
Time between HIV diagnosis and EBUS-TBNA (years)	14 ± 11
Antiretroviral initiated at the time of EBUS-TBNA	11 (73%)
Viral load < 20 copies/ml	7 (47%)
**Chest computed tomography**	
**Lymphnode stations involved**	
2	3 (20%)
4	8 (53%)
6	1 (7%)
7	7 (47%)
10	2 (13%)
11	4 (27%)
12	1 (7%)
Lymphnode size (cm)	2.25 ± 0.62
**PET/CT results**	
SUV max	7.6 ± 2.8

Mean ± standard deviation, number (%).

### Clinical and radiological data

The most frequent symptoms leading to the diagnosis of enlarged thoracic lymphadenopathy were fever (67%), sweats (40%), hemoptysis (33%), and weight loss (33%). The most frequently involved mediastinal node stations were stations 4 (7/15, 47%), 7 (7/15, 47%), and 11 (4/15, 27%). The most common associated radiological features were: lung consolidation (4/15, 47%), and lung mass (3/15, 20%). Extra-thoracic radiological signs were extra-thoracic lymphadenopathy (6/15), adrenal mass (2/15), splenic nodules (2/15), osteolysis (2/15). Twelve patients underwent a PET/CT, revealing FDG-avid mediastinal lymphnodes in all patients, with a median SUV max of 7.6 ± 2.8.

### Endoscopy and EBUS-TBNA results

Endoscopy and EBUS-TBNA results are summarized in Table 2. Bronchoscopic alveolar lavage (BAL) was performed in 6 patients (40%), showing alveolitis with macrophages (n = 4), polynuclear neutrophils (n = 1), or lymphocytes (n = 1). During EBUS-TBNA, a mean of 4.9 ± 0.9 punctures was performed. Subcarina and paratracheal stations were the most common sampled sites in respectively 47% and 40% of our patients. There were no complications during the procedure, except one bronchospasm leading to anticipated interruption of the exam.

**Table 2:** EBUS-TBNA procedures and results.

Characteristics	Patients n = 15
**Lymphnode stations sampled**	
2	1 (7%)
4	6 (40%)
7	7 (45%)
10	3 (20%)
11	3 (20%)
**Histological results**	
Gigantocellular epithelioid granuloma	3 (20%)
with caseous necrosis	1 (7%)
without caseous necrosis	2 (13%)
Epidermoid carcinoma	1 (7%)
Non-small cell carcinoma	1 (7%)
Adenocarcinoma	1 (7%)
Hyaline vascular Castleman disease	1 (7%)
Anthracosic nodule	
**Microbiological results**	
Positive smear for acid-fast bacilli:	1 (7%)
Positive culture	
*Mycobacterium tuberculosis*	2 (13%)
*Mycobacterium avium-intracellulare*	3 (20%)
**Xpert MTB/RIF**	
Positif for *Mycobacterium tuberculosis*	2 (13%)
Negatif for rifampin resistance mutation	2 (13%)
**Side effects**	
Bronchospasm	1 (7%)
**Diagnosis after EBUS-TBNA**	
Tuberculosis	2 (13%)
Infection à *Mycobacterium avium-intracellulare*	3 (20%)
Adenocarcinoma	1 (7%)
Epidermoid carcinoma	2 (13%)
Non-small cell carcinoma	1 (7%)
Castelman disease	1 (7%)
Sarcoidosis	1 (7%)
Professional dustiness	1 (7%)
**Surgical procedures following EBUS-TBNA**	
Mediastinoscopy	1 (7%)
Thoracoscopy	1 (7%)

Mean ± standard deviation, number (%).

Adequate lymphoid tissue was obtained in all patients. A positive histological result of malignancy was found in 4 patients, showing epidermoid cancer (n = 2), adenocarcinoma (n = 1), non-small cell carcinoma (n = 1). Histological analysis confirmed Castleman’s disease in one case and professional dustiness in one case. Gigantocellular epithelioid granulomas were found in 4 patients with caseous necrosis in 3 patients. Microbiological analysis revealed positive mycobacterial culture for *M. tuberculosis* in 2 patients and for *M. avium-intracellulare* in 3 patients. Retained diagnosis was sarcoidosis in one patient, showing negative mycobacterial culture. This patient underwent mediastinoscopy, which confirmed the diagnosis of sarcoidosis and the absence of mycobacterial infection. Finally, EBUS-TBNA established a specific diagnosis in 12 patients (80%). In 3 patients, histological analysis revealed normal lymphatic tissue. One patient benefited from a lung segmentectomy by open thoracotomy leading to the diagnosis of tuberculosis. Two others patients became asymptomatic with antiretroviral therapy alone. Follow-up for more than 1 year revealed no additional disease and they were assumed to have non-specific lymphadenitis. In our case-series, EBUS-TBNA diagnostic sensitivity, specificity and accuracy were respectively 92%, 100% and 93%.

## Discussion

In our settings, EBUS-TBNA was rarely performed in HIV-infected patients with thoracic lymphadenopathy, even if its use increased since 2015. In our case-series, the procedure was safe and accurate in more than 90% of cases.

Only three studies have previously reported EBUS-TBNA in HIV-infected patients. They were all conducted in countries of tuberculosis high or intermediate burden: Mexico, Singapour, and India^9–11^. The WHO’s global tuberculosis database last update estimates incidence of tuberculosis to be respectively 23, 47 and 199 per 100,000 population per year in Mexico, Singapour and India^13^. To our knowledge, our case-series is the first to analyse EBUS-TBNA performance in HIV-infected patients in a tuberculosis low-incidence area. Last estimation of tuberculosis incidence in France is 9 per 100,000 population per year, and even lower in Northern area of France.

Despite the tuberculosis low-incidence in our area, tuberculosis was diagnosed in 20% of our patients, confirming the still high-burden of this infection in HIV-infected patients. Previously published studies reported tuberculosis in 29 to 77% of HIV-infected patients who underwent EBUS-TBNA^9–11^. If we considered all mycobacterial infections, including tuberculosis and atypical mycobacterial infections, these infections represented the most frequent diagnosis in 40% of our patients. Similarly, Han et al. and Sanchez-Cabral et al. reported frequent infections with non-tubercular mycobacteria^9,10^.

In our study, lung cancer was the second most frequent diagnosis, established in 4 patients (27%). In the era of combination antiretroviral therapy, a high prevalence of non-HIV related malignancy has been reported in HIV-infected patients with mediastinal lympadenopathy, even if lymphoma remained the most common neoplasia^14^. None of our patients was diagnosed with lymphoma while it accounted for half of the neoplasms in previously published studies evaluating EBUS-TBNA in HIV-infected patients^9–11^. Of note our patients had higher median CD4 cells count than patients recruted in those studies. While 73% of our patients were receiving antiretroviral therapy, only 18% of patients have initiated antiretroviral therapy in the only study which made this data available^11^. Another reason for the lack of diagnosis of lymphoma in our study could be the choice of performing a medianoscopy in case of suspicion of lymphoma rather than EBUS-TBNA. Many clinicians still have concern that the small size of the samples obtained with EBUS-TBNA would not be sufficient to properly determine the pathologic subtype, essential for the management of lymphoma^15^. The frequency of malignancy justified lymphadenopathy sampling for histological analysis whenever possible in HIV-infected patients with mediastinal lymphadenopathy.

Accuracy of EBUS-TBNA has been established for most of the affections which can account for mediastinal lymphadenopathy in HIV-infected patients. Others diagnosis established after EBUS-TBNA performance in our cohort were sarcoidosis, Castleman’s disease and professional dustiness. Each of these diseases were diagnosed in one of our patients. The variety of diagnosis is another justification for etiological investigation of mediastinal lymphadenopathy in HIV-infected patients in tuberculosis low-incidence area.

To date, mediastinoscopy is still considered the gold standard for diagnosis of mediastinal lymphadenopathy. But compared with mediastinoscopy, EBUS-TBNA can be performed using moderate sedation. None of our patients needed general anesthesia. It is a safe procedure, even in HIV-infected patients with low CD4 cell count. In our case-series, one patient out of fifteen (7%), experienced moderate bronchospasm. Sanchez-Cabral et al. reported one pneumomediastinum (2%)^10^. No mediastinis had been described after EBUS-TBNA performance in HIV-infected patients. In a large published EBUS-TBNA series, including 1891 patients, only a very low rate (0.74%) of post-procedure minor complications were described, with no major complication^16^. EBUS-TBNA has the advantage over mediastinoscopy to routinely access posterior mediastinal (station 7) and hilar lymph nodes (stations 10 and 11). In our cohort, station 7 was the most common sampled site.

EBUS-TBNA was first validated for mediastinal staging in patients with lung cancer^6^. It was then used for diagnosis confirmation of various pathologies with mediastinal lymphadenopathies including tuberculosis, sarcoidosis and lymphoma^8,9,17^. EBUS-TBNA accuracy was high in selected patients with a high pre-test probability of a specific diagnosis. In a study including 158 patients with high clinical suspicion of mediastinal tuberculous lymphadenitis, EBUS-TBNA demonstrated a diagnostic accuracy of 98% using culture and histological results. Similarly, the diagnostic accuracy of EBUS-TBNA was 96% in patients with suspicion of lymphoma raised because of history of lymphoma, known lymphoma elsewhere, or clinical and radiological compatible presentation^17^.

But the overall diagnostic yield of EBUS-TBNA performed for intrathoracic lymphadenopathy in non-HIV patients dropped to 50–60%^18,19^. All 3 previously published studies performed in HIV infected patients reported higher global accurancy of EBUS-TBNA, ranging from 60–89%^9–11^. The author attributed these results to the high frequency of infections, especially tuberculosis. Tuberculosis can be diagnosed with multiple modalities including smear, culture and nucleic acid amplification^20^.

As the authors assumed before the initiation of this study, the accuracy of EBUS-TBNA in HIV patients, even in a TB low burden area was higher than the overall accuracy observed in non-HIV patients^18,19^. Our study confirmed the clinical significance of mediastinal lymphadenopathy in HIV-infected patients in the era of combination antiretroviral therapy and the justification of invasive sampling performance if necessary to reach a specific diagnosis. In our case-series, EBUS-TBNA diagnostic sensitivity, specificity and accuracy were respectively 92%, 100% and 93%. Among our patients, 80% underwent a PET/CT prior EBUS-TBNA performance revealing FDG-avid mediastinal lymphnodes in all patients. Choice of sampling sites guided by PET/CT probably helped to improve EBUS-TBNA diagnostic yield^21^. Use of more invasive procedures was avoid in the majority of our patients. Mediastinoscopy was performed in one patient, confirming sarcoidosis diagnosis already retained by EBUS-TBNA results. Thoracotomy was performed in one patient with associated pulmonary consolidation for whom EBUS-TBNA results were negative. It led to the diagnosis of tuberculosis.

Our study as several limits. Because of its retrospective nature, the patients who benefited from EBUS-TBNA were selected by the prescribers as being the most likely to take advantage of the procedure. But others patients could have been good candidates for EBUS-TBNA performance. The number of patients included was low. Analysis on a larger sample of patients is required to confirm the high yield of EBUS-TBNA in HIV-infected patients with thoracic lymphadenopathy. But, the limited number of patients in our study reflected the need to promote EBUS-TBNA in HIV setting and improve knowledge about this technique among infectious disease specialists.

In conclusion, our study conducted in the setting of tuberculosis low-incidence confirmed the safety and high diagnostic yield of EBUS-TBNA in the investigation of mediastinal lymphadenopathy in HIV-infected patients. EBUS-TBNA may replace more invasive procedures such as mediastinoscopy as first-line technique.

## Data Availability

Data are available from the authors upon request (contact: Dr Agnès Meybeck, agnesmeybeck@yahoo.fr).

## References

[1] Jasmer RM (2002). Clinical and radiographic predictors of the etiology of computed tomography-diagnosed intrathoracic lymphadenopathy in HIV-infected patients. J. Acquir. Immune Defic. Syndr..

[2] Fishman JE, Sagar M (1999). Thoracic lymphadenopathy in HIV patients: Spectrum of disease and differential diagnosis. AIDS Patient Care STDS..

[3] Chou SH (2014). Thoracic diseases associated with HIV infection in the era of antiretroviral therapy: Clinical and imaging findings. Radiographics.

[4] Harkin TJ (1998). Transbronchial needle aspiration (TBNA) in patients infected with HIV. Am. J. Respir. Crit. Care Med..

[5] HAS (French National Authority for Health - Haute Autorité de santé). Endobronchial ultrasound-guided transbronchial needle aspiration. ISBN number: 978–2–11–139050–8. Available at:https://www.has-sante.fr/portail/jcms/c1770048/fr/echo-endoscopie-bronchique-avec-ponction-transbronchique-a-l-aiguille. Last access: 20th January 2020.

[6] Fujiwara T (2010). The utility of sonographic features during endobronchial ultrasound-guided transbronchial needle aspiration for lymphnode staging in patients with lung cancer: A standard endobronchial ultrasound image classification system. Chest.

[7] Navani N (2011). Utility of endobronchial ultrasound-guided transbronchial needle aspiration in patients with tuberculous intrathoracic lymphadenopathy: A multicentre study. Thorax.

[8] Gupta D (2014). Endobronchial ultrasound-guided transbronchial needle aspiration vs. conventional transbronchial needle aspiration in the diagnosis of sarcoidosis. Chest.

[9] Han AY, Tan AH, Koh MS (2015). Utility of endobronchial ultrasound-guided transbronchial needle aspiration in diagnosis of intrathoracic lymphadenopathy in patients with human immunodeficiency virus infection. Biomed. Res. Int..

[10] Sanchez-Cabral O (2017). Usefulness of endobronchial ultrasound in patients with human immunodeficiency virus infection and mediastinal lymphadenopathy. Respiration..

[11] Prasad KT (2018). Utility of endobronchial ultrasound-guided transbronchial needle aspiration in HIV-infected patients with undiagnosed intrathoracic lymphadenopathy. Lung India..

[12] Pugliese P (2003). NADIS 2000, development of an electronic medical record for patients infected by HIV, HBV and HCV. Press. Med..

[13] World Health Organization. Global tuberculosis report 2019. (2019).

[14] Alcada J (2014). High prevalence of malignancy in HIV-positive patients with mediastinal lymphadenopathy: A study in the era of antiretroviral therapy. Respirology.

[15] Horiana B, Grosu MD (2018). EBUS-TBNA for the diagnosis of lymphoma. Time to give in?. J. Bronchol. Intervent. Pulmonol..

[16] Guarize J (2018). Endobronchial ultrasound transbronchial needle aspiration in thoracic diseases: Much more than mediastinal staging. Respir. J Can.

[17] Kennedy MP (2008). Endobronchial ultrasound-guided transbronchial needle aspiration in the diagnosis of lymphoma. Thorax.

[18] Dhooria S (2017). Diagnostic yield and complications of EBUS-TBNA performed under bronchoscopist-directed conscious sedation: Single center experience of 1004 subjects. J. Bronchol. Interv. Pulmonol..

[19] Ost DE (2011). Diagnostic yield of endobronchial ultrasound-guided transbronchial needle aspiration: Results of the AquiRE broncoscopy registry. Chest.

[20] Chhajed PN (2019). EBUS-TBNA in the rapid microbiological diagnosis of drug-resistant mediastinal tuberculous lymphadenopathy. ERJ Open Res..

[21] Gan Q, Stewart JM, Valik E, Eapen G, Caraway NP (2019). Cytologic evaluation of positron emission tomography-computed tomography-positive lymph nodes sampled by endobronchial ultrasound-guided transbronchial needle aspiration: Experience at a large cancer center. Arch. Pathol. Lab. Med..

